# Case Report: Clinicopathologic and molecular characteristics of prostate adenocarcinoma brain metastases despite disproportionately low PSA levels: a four-patient case series and narrative review

**DOI:** 10.3389/fonc.2026.1905004

**Published:** 2026-10-13

**Authors:** Roshmita Bardhan, Zin W. Myint, Harit Kapoor, Insija Selene

**Affiliations:** 1 Department of Internal Medicine, The Jewish Hospital-Mercy Health, Cincinnati, OH, United States; 2 Internal Medicine/Medical Oncology Division, University of Kentucky, Kentucky, KY, United States; 3 Radiology, University of Kentucky, Kentucky, KY, United States; 4 Internal Medicine/medical Oncology Division, University of Kentucky, Kentucky, KY, United States

**Keywords:** androgen deprivation, brain metastases, molecular profiling, prostate adenocarcinoma, prostate specific antigen

## Abstract

Brain metastases (BM) from prostate cancer (PCa) are rare, typically occurring in a small subset of patients with advanced disease or tumors with neuroendocrine differentiation. Diagnosis can be particularly challenging when prostate-specific antigen (PSA) levels remain low or undetectable. We describe four patients with prostate adenocarcinoma who developed brain metastases despite low or modestly elevated PSA levels relative to disease burden(<0.01 to 6.5 ng/ml). All patients underwent surgical resection followed by focal stereotactic radiosurgery and androgen deprivation therapy, with androgen receptor pathway inhibitors used in selected cases. Molecular profiling was available for three patients and demonstrated heterogeneous genomic findings, including PTEN alteration (Case 1), CDK4 amplification (Case 2), and an ATM mutation (Case 3), while testing was unavailable in Case 4. No pathogenic TP53 or RB1 alterations were identified in the profiled tumors. Two patients achieved durable biochemical and radiographic complete remission, one remained in remission before dying of an unrelated cause, and one developed biochemical recurrence without radiographic progression. These findings highlight the biological heterogeneity of central nervous system involvement in prostate cancer and emphasize that brain metastases should be considered even in patients with low PSA levels. Further studies are needed to better define the molecular determinants and biomarkers associated with this uncommon presentation.

## Introduction

Prostate cancer brain metastases (PCBM) are rare, occurring in approximately 1-2% of cases, and are typically associated with advanced castration resistant disease or with neuroendocrine differentiation (1–3). Neurological symptoms are often the first indication, yet diagnosis may be delayed because BM is not routinely considered in men with metastatic Pca (1, 2). Low PSA levels further complicate evaluation, as they may reflect dedifferentiation, reduced androgen receptor (AR) dependence, or alternative lineage programs rather than low disease burden (4–7). Recent advances in systemic therapy have substantially prolonged survival in metastatic PCa, leading to increasing recognition of uncommon metastatic sites, including the central nervous system (8). Xue et al. reported a substantial increase in the burden of urological cancers in China between 1990 and 2021, with 266,887 incident cases and 159.5 million prevalent cases in 2021; notably, prostate cancer surpassed bladder cancer as the leading subcategory, with the greatest increases in incidence seen among those aged 55 and older (9). Similarly, the global burden of prostate cancer continues to rise, with the annual number of new cases projected to increase from approximately 1.4 million in 2020 to 2.9 million by 2040, largely driven by population aging and increased life expectancy (10). These epidemiologic trends are expected to increase the recognition of uncommon metastatic presentations, including central nervous system involvement.

Large-scale genomic studies have demonstrated enrichment of TP53, RB1, androgen receptor (AR), and PI3K/AKT/PTEN pathway alterations in metastatic PCa compared with primary tumors (11, 12). More recently, retrospective molecular profiling studies of PCBM have identified recurrent TP53 alterations, homologous recombination repair (HRR) gene alterations, activation of the PI3K/AKT/PTEN pathway, and increased genomic complexity, suggesting that CNS dissemination is frequently associated with biologically aggressive disease (13–15). However, these studies are limited by the rarity of PCBM, resulting in small retrospective cohorts, heterogeneous sequencing platforms, limited availability of matched primary and metastatic tumor samples, and scarce correlation between molecular findings and clinical outcomes. Consequently, the molecular determinants of CNS tropism remain incompletely understood (13–15).

We report four cases of prostate adenocarcinoma presenting with brain metastases despite low or modestly elevated PSA levels relative to disease burden and characterize their clinicopathologic features, molecular findings where available, treatment and clinical outcomes, highlighting the biological heterogeneity and diagnostic challenges of this rare presentation.

## Cases series

### Case 1

A 67-year-old male with no prior history of metastatic prostate cancer presented with multiple falls. Brain MRI demonstrated a right cerebellar mass and an additional left temporal lobe lesion, while CT of the chest revealed multiple bilateral pulmonary nodules suspicious for metastatic disease. His PSA at presentation was 6.5 ng/mL. The patient underwent right cerebellar craniotomy, and histopathologic examination confirmed metastatic prostate adenocarcinoma.

Comprehensive molecular profiling of the resected brain metastasis using the Caris Molecular Intelligence^®^ platform demonstrated a likely pathogenic PTEN alteration, ERG fusion, positive AR expression (2+, 90%), microsatellite stable (MSS) status, proficient mismatch repair (MMR), low genomic loss of heterozygosity (LOH), and a low tumor mutational burden (3 mutations/Mb).

Following surgical resection, the patient underwent HyperArc stereotactic radiosurgery (3000 cGy in 5 fractions to PTV1 and 2500 cGy in 5 fractions to PTV2 using 6X flattening filter-free beams) and subsequently initiated androgen deprivation therapy (ADT) plus apalutamide. At 4.5 years of follow-up, his PSA remained <0.01 ng/mL, and serial brain MRI and CT imaging demonstrated no evidence of recurrent intracranial disease or progression of pulmonary metastases. (**Figures 1**, **2**)

**Figure 1:**
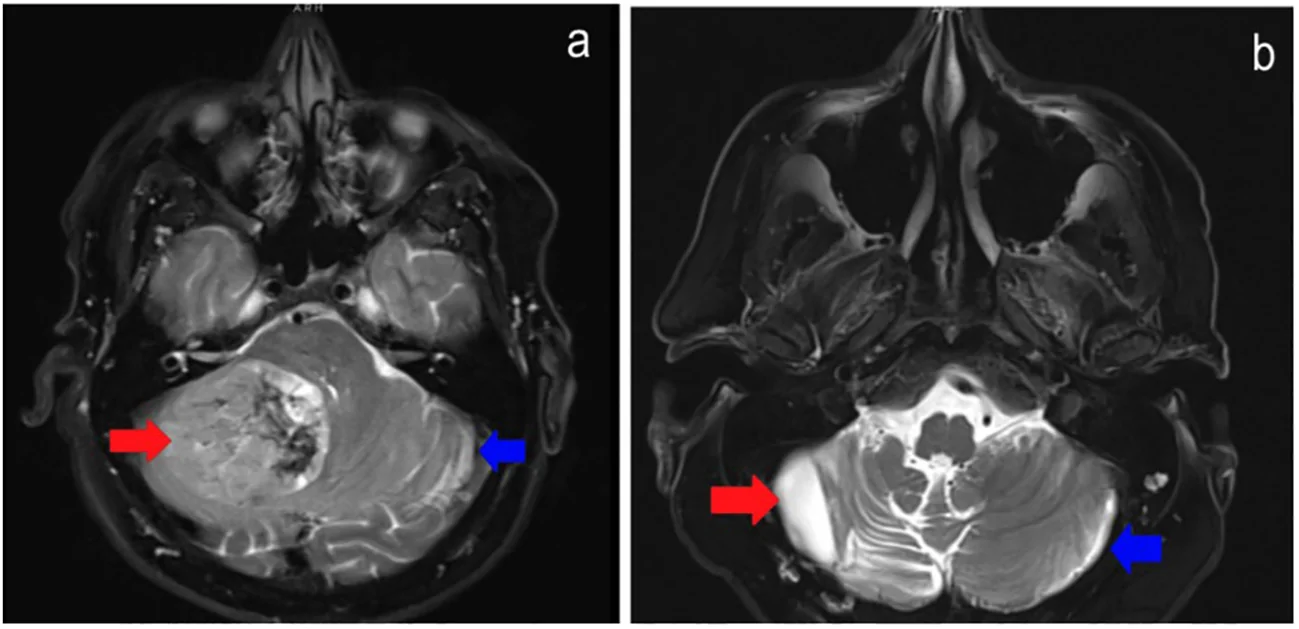
Pretreatment [Image **(a)**]- MRI brain revealing right cerebellar hemisphere (red arrow) and an additional lesion present within the posterior aspect of the left temporal lobe (blue arrow). Post-treatment [Image **(b)**]- Postsurgical changes status post right cerebellar resection seen in the MRI brain (red arrow). No new areas of suspicious enhancement. Previously noted left temporal periventricular enhancement is less conspicuous on imaging (blue arrow).

**Figure 2:**
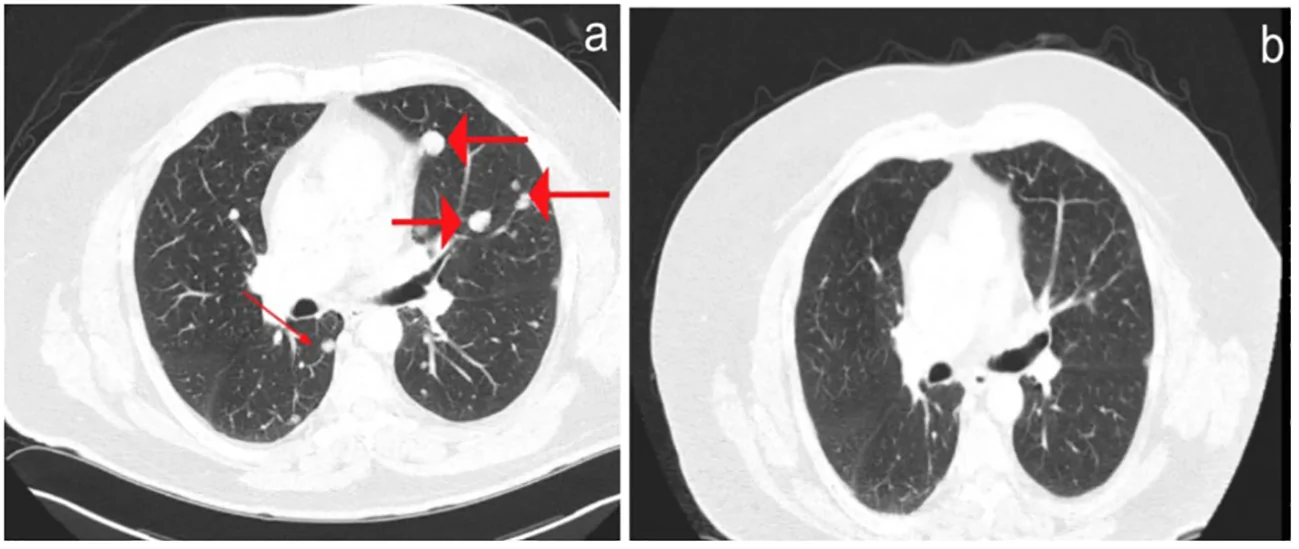
Pre-treatment [Image **(a)**]- Multiple lung metastases bilaterally seen in CT chest. Post-treatment [Image **(b)**]- Negative for cancer progression. Emphysematous changes noted in CT Chest.

### Case 2

A 65-year-old male with high-risk localized prostate adenocarcinoma (pT3bN1, Gleason score 4 + 4 = 8) underwent radical prostatectomy followed by salvage radiation therapy and short-course ADT, achieving an undetectable PSA(<0.01). Seven years later, he presented with new-onset seizures. Brain MRI demonstrated a solitary cystic frontal lobe mass (**Figure 3**), while systemic staging showed no extracranial disease. Surgical resection confirmed metastatic prostate adenocarcinoma. Comprehensive molecular profiling using foundation one of the resected brain metastasis demonstrated CDK4 amplification with an intermediate TMB (10 mutations/Mb). Following adjuvant Gamma Knife stereotactic radiosurgery, his PSA increased to 0.05 ng/mL, accompanied by enlarged hilar lymph nodes, and he was restarted on ADT. He achieved biochemical and radiographic complete remission and remained free of recurrent intracranial or systemic disease for an additional 5 years. The patient subsequently died of septic shock, with a PSA remaining <0.01 ng/mL at the time of death. Image

**Figure 3:**
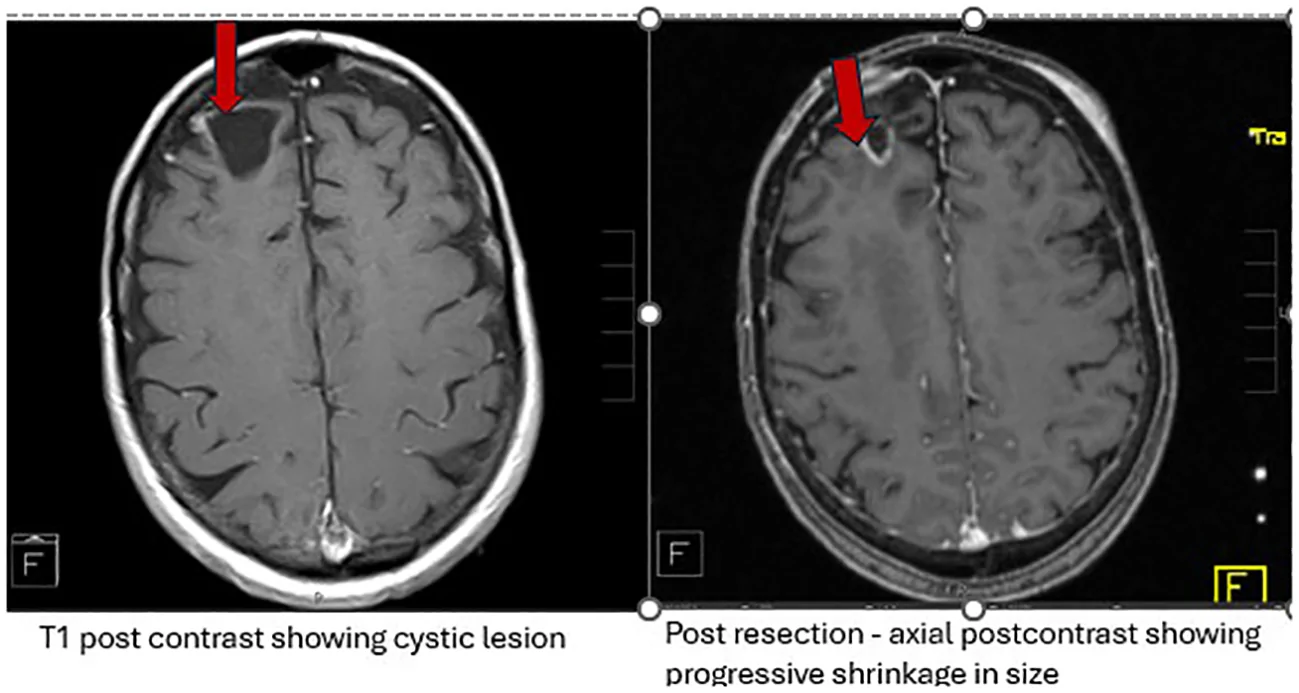
Pre-treatment and Post treatment MRI.

### Case 3

A 70-year-old male with high-risk localized prostate adenocarcinoma (pT3aN0, Gleason score 4 + 5 = 9) underwent radical prostatectomy followed by salvage radiation therapy, achieving a PSA nadir of 0.38 ng/mL. Twelve months later, his PSA increased to 1.28 ng/mL. prostate -specific membrane antigen(PSMA) PET/CT demonstrated focal uptake in the left occipital region, and brain MRI revealed a solitary 8.5 × 7 × 9.5 mm (**Figure 4**) enhancing lesion. Surgical resection confirmed metastatic prostate adenocarcinoma. Comprehensive molecular profiling of the resected brain metastasis using the Caris Molecular Intelligence^®^ platform identified a pathogenic ATM mutation (p.R2763*), TMPRSS2::ERG fusion, high LOH, positive AR expression (3+, 95%), MSS, proficient MMR, and low TMB (3 mutations/Mb), Following adjuvant Gamma Knife stereotactic radiosurgery, his PSA increased to 5.42 ng/mL, and repeat PSMA PET/CT demonstrated metastases to the left internal and external iliac lymph nodes without recurrent intracranial disease (**Figure 5**). He was treated with ADT plus apalutamide, achieving durable biochemical and radiographic complete remission. At 24 months of follow-up, his PSA remained <0.01 ng/mL, with no evidence of recurrent intracranial or systemic disease.

**Figure 4:**
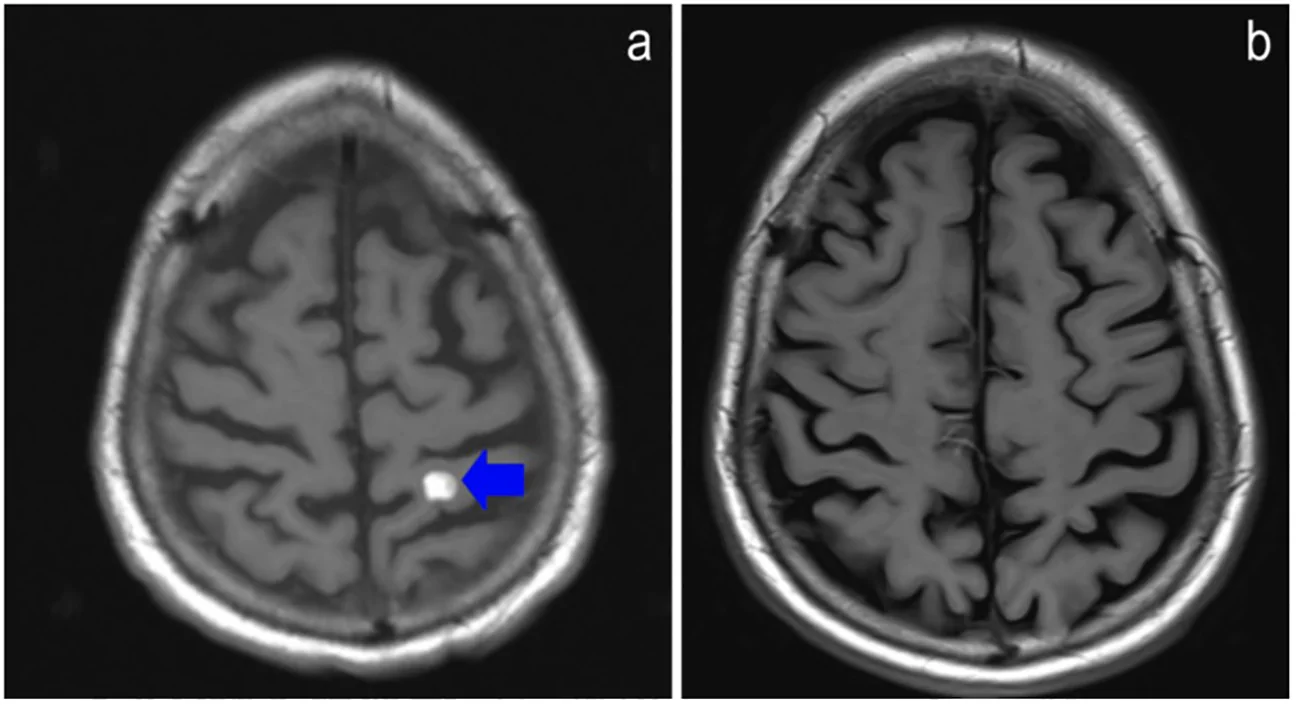
Pre-treatment [Image **(a)**]- MRI brain revealing peripherally enhancing focus in left occipital region, concerning for metastasis (blue arrow). Post-treatment [Image **(b)**]- Negative for metastasis. Status post left occipital craniotomy.

**Figure 5:**
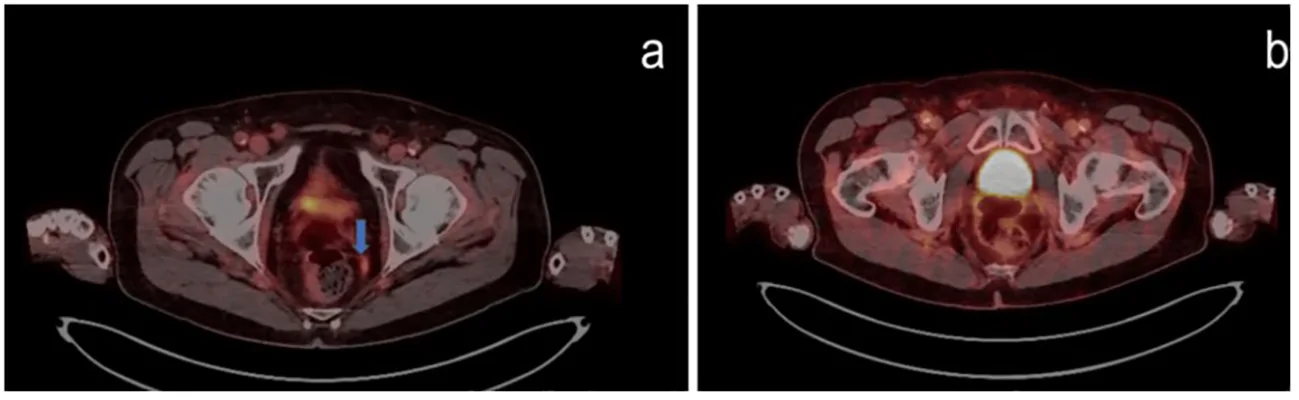
Pre-treatment [Image **(a)**] -PET scan reveals interval development of small, PSMA avid left internal and external iliac lymph nodes, highly suspicious for nodal metastatic disease (blue arrow). Post-treatment [Image **(b)**]- No suspicious focal PSMA avidity in the prostate bed, regional lymph node metastasis or distant metastatic disease.

### Case 4

An 82-year-old male with low-risk localized prostate adenocarcinoma treated with brachytherapy presented with a near-syncopal episode 17 years after diagnosis. His PSA was 3.65 ng/mL. Brain MRI demonstrated two enhancing left parietal lesions measuring 3.0 × 3.9 × 4.0 cm and 3.7 cm (**Figure 6**), while fluciclovine (Axumin) PET/CT showed no extracranial metastatic disease. Left craniotomy confirmed metastatic prostate adenocarcinoma with ductal differentiation. Comprehensive molecular profiling was not performed. The patient underwent adjuvant Gamma Knife stereotactic radiosurgery followed by ADT with leuprolide. His PSA decreased to 0.14 ng/mL. At 4 years of follow-up, brain MRI and PSMA PET/CT demonstrated no evidence of recurrent intracranial or systemic disease. Although his PSA subsequently increased to 2.56 ng/mL, there was no radiographic evidence of disease progression.

**Figure 6:**
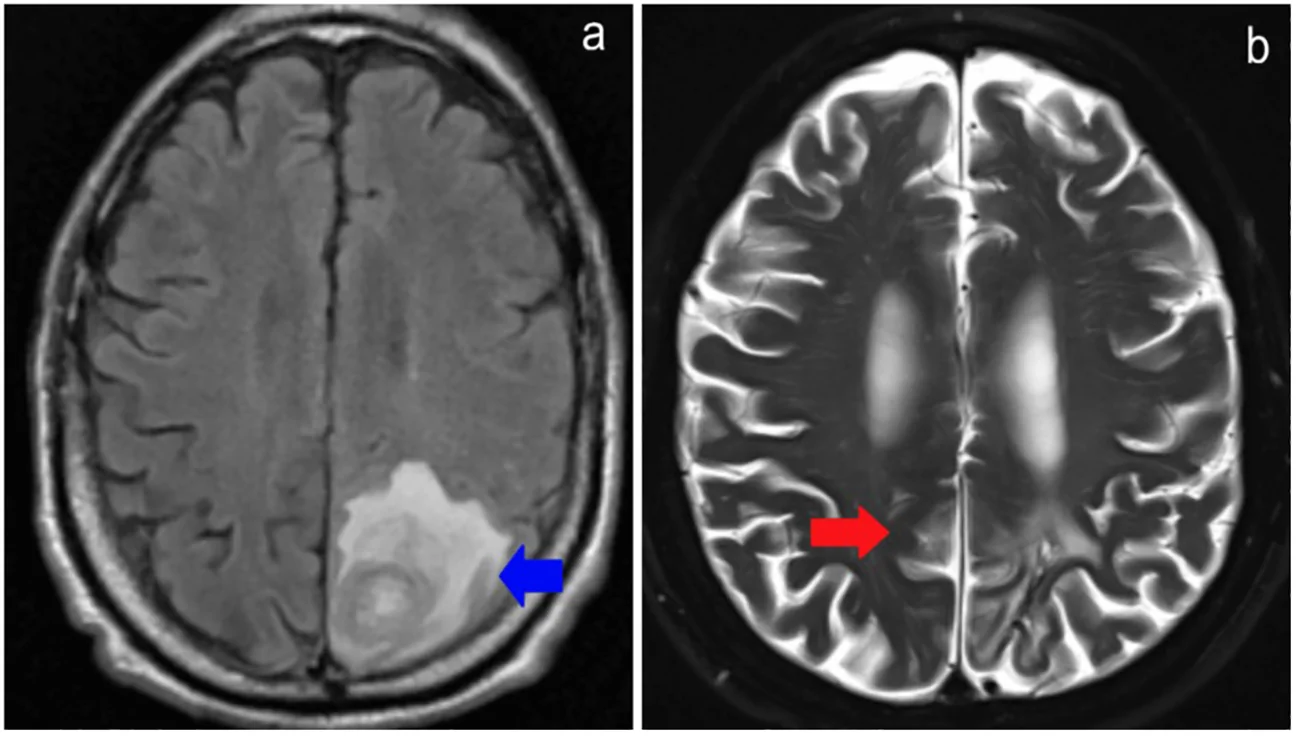
Pre-treatment [Image **(a)**] - Enhancing parietal lesion. Post- treatment [Image **(b)**]- Resection cavity within the left parietal lobe, stable from prior study. No new nodular or masslike postcontrast enhancement.

## Discussion

BM from prostate cancer remains uncommon and has historically been associated with late-stage, heavily pretreated disease characterized by widespread systemic involvement (1–3). Prior clinicopathologic series have described BM in patients with neuroendocrine or small-cell variants or high-grade adenocarcinoma, with outcomes generally poor and median survival measured in months despite radiotherapy and systemic treatment (4–6). In contrast, BM arising from pure adenocarcinoma is less frequently reported and biologically heterogeneous, ranging from isolated CNS disease to high-Gleason score tumors in patients with extensive systemic involvement (1, 16–19).

A defining feature of our cohort was the relatively low PSA values at the time of brain metastasis diagnosis, ranging from undetectable to 6.5 ng/mL. Although one patient had a PSA of 6.5 ng/mL, these values are disproportionately low considering the presence of intracranial metastatic disease, highlighting that PSA may underestimate disease burden in selected patients with biologically aggressive tumors. Low PSA is increasingly recognized as a hallmark of biologically aggressive prostate cancer rather than indolent disease (5, 7). High-grade tumors, lineage plasticity, attenuation of androgen receptor (AR) signaling, and certain molecular alterations result in disproportionately low PSA expression despite significant tumor burden (4–7). Reports of BM occurring with low or normal PSA are rare and are limited to case reports (18–22). Lungulescu et al. described a patient with solitary brain metastasis and normal PSA harboring a PALB2 mutation, while Cheng et al. recently reported multiple brain metastases developing after radical prostatectomy and androgen deprivation therapy despite low psa (18, 19). These reports, together with our series, demonstrate the heterogeneous clinical presentation of prostate cancer brain metastases. Unlike previous reports, our study includes four patients with prolonged follow-up and molecular characterization of three resected brain metastases, demonstrating diverse genomic alterations rather than a single recurrent molecular subtype. Collectively, these findings suggest that relatively low PSA should not preclude evaluation for central nervous system metastases in patients with compatible neurological symptoms and support comprehensive molecular profiling whenever feasible. Prior cohorts of “low-PSA, high-risk” prostate cancer have demonstrated inferior cancer-specific outcomes, highlighting PSA’s limitations as a surrogate for disease burden or biological aggressiveness in certain metastatic phenotypes (5–7).

Advances in imaging, particularly PSMA PET, have improved the detection of recurrent and metastatic prostate cancer, even at very low PSA levels. Detection rates remain meaningful at PSA <0.2 ng/mL, well below sensitivity thresholds of conventional imaging (20–22). Case reports and small series described incidental detection of brain or skull metastases on PSMA PET/CT, as well as identification of intracranial lesions initially overlooked on MRI. These findings suggest that PSMA PET, when combined with dedicated brain MRI, may provide complementary information and enhance detection of occult CNS involvement in patients with atypical symptoms or biochemical–clinical discordance (20–22).

The molecular mechanisms underlying BM in PCa remain incompletely understood. Genomic analyses have implicated alterations in TP53 and RB1, PTEN, and homologus recombination repair (HRR)genes (e.g., BRCA2, ATM, PALB2), as well as dysregulation of AR signaling, the PI3K/AKT pathway, and cell-cycle regulators in CNS dissemination (13–15, 23–25). Inactivation of TP53 and RB1, particularly concurrent loss, has been strongly associated with lineage plasticity and neuroendocrine transformation, which are recognized features of aggressive prostate cancer and have been implicated in metastatic progression, including CNS involvement (23–25). In a comprehensive genomic comparison across multiple metastatic sites, Necchi et al. demonstrated that brain metastases exhibited the greatest genomic complexity, with an average of eight genomic alterations per tumor, enrichment of PTEN alterations, and a substantially higher prevalence of microsatellite instability (MSI) than other metastatic sites (14). Using targeted sequencing, Rodríguez-Calero et al. reported HRR alterations in approximately 19.6% of prostate cancer brain metastases, suggesting that a subset of patients may benefit from PARP inhibitor therapy (15). Epigenetic changes have further implicated hypomethylation of GABRB3, CLDN4, and CLDN8, together with hypermethylation in SPOP-mutant tumors, supporting a role for epigenetic regulation in CNS tropism (25). Armenia et al. further demonstrated marked genomic heterogeneity among metastatic prostate cancers, including enrichment of mutations in epigenetic regulators in ETS fusion-negative tumors (6). Reported frequencies of individual alterations, particularly BRCA2 (0–20%), vary considerably across studies, likely reflecting differences in cohort size, tissue source, sequencing platforms, sequencing depth, and the use of targeted gene panels versus whole-genome sequencing (13–15).

Consistent with the molecular heterogeneity reported in previous studies, molecular profiling of three resected brain metastases in our cohort demonstrated heterogeneous genomic alterations, including a PTEN alteration in one patient, CDK4 amplification with intermediate tumor mutational burden in another, and a pathogenic ATM mutation with high genomic loss of heterozygosity and TMPRSS2::ERG fusion in a third. No pathogenic TP53 or RB1 alterations were identified among the profiled cases. These findings emphasize the molecular heterogeneity of prostate cancer brain metastases rather than a single genomic pathway underlying CNS dissemination. Although limited by the small sample size and incomplete molecular profiling across all patients, our series underscores the value of comprehensive molecular characterization in patients with atypical metastatic presentations.

Management of prostate cancer BM requires a multidisciplinary approach incorporating both local and systemic therapies (1–3, 8). Local treatment options include surgical resection and stereotactic radiosurgery, with whole-brain radiotherapy reserved for select cases. Systemic therapy follows standard paradigms for advanced prostate cancer and may include androgen deprivation therapy, androgen receptor pathway inhibitors, chemotherapy, and in molecularly selected patients, targeted therapies such as PARP inhibitors (8, 26).

Although survival following BM diagnosis has historically been limited, emerging data suggest more favorable outcomes in patients with solitary or oligometastatic CNS disease who are candidates for aggressive local therapy and who maintain systemic disease control. Consistent with this observation, all patients in our cohort underwent focal CNS-directed therapy in combination with ADT, and several received ARPIs, achieving durable biochemical and radiographic complete remission.

## Conclusion

PCBM remains rare and poorly understood, with diagnosis often complicated by low PSA levels. This case series highlights the clinicopathologic and molecular heterogeneity of prostate cancer brain metastases, which may occur despite low or modestly elevated PSA levels that appear disproportionate to the extent of metastatic disease. Clinicians should therefore maintain a high index of suspicion when new neurological symptoms arise, regardless of PSA level. Comprehensive molecular profiling may improve understanding of the biological mechanisms underlying CNS dissemination and identify clinically relevant biomarkers that can guide risk stratification and therapeutic decision making.

This series is limited by its small sample size, retrospective design, and single-institution experience, which restrict the generalizability of our findings. Molecular profiling was unavailable for one patient, and testing was performed using different sequencing platforms across cases, precluding direct comparison of genomic findings. Additionally, the absence of matched primary tumor sequencing limits our ability to determine whether the identified alterations were acquired during metastatic progression or were already present in the primary tumor. Larger prospective studies are needed to better define the molecular landscape of prostate cancer brain metastases and its implications for patient management.

## Patient perspective

Case 1: After experiencing several unexpected falls, I was sad to learn that the prostate cancer had spread to my brain and lungs. At that time, I was not sure what the future would hold. The surgery and treatments were long and difficult. The hormone therapy brought constant fatigue and hot flashes. I also lost so much of my strength, which made simple and everyday tasks harder than before. My doctors took the time to explain everything and helped me along the way. I am doing better now and I am thankful for the treatment.

Case 2: Patient perspective unavailable due to patients death.

Case 3: After going through surgery and radiation for my prostate cancer, I was finally getting back to my routine. But when the doctors told me they had found a spot in my brain just a few months later, I was worried about having to face cancer all over again. The medicines helped treat it, but they came with their own problems, like trouble sleeping, mood swings, and feeling weaker than I used to, which made it harder to stay active. My family and medical team helped me get through it. I am glad my PSA is now undetectable. I hope I can put this behind me.

Case 4: It had been 17 years since I was treated for prostate cancer. Then I had a spell where I nearly passed out, which left me confused. I did not know why it had happened, because everything had seemed fine before that. A little later, the doctors told me they had found concerning areas in my brain. I was surprised to learn that it was related to cancer after all these years. The treatment helped keep things under control, but the hormone shots left me feeling slower. I also had muscle aches that I noticed more each day. Knowing that the cancer has not come back is reassuring. I still worry about what the future holds, since my cancer returned after so long.

## Data Availability

The data supporting the findings of this case series are derived from individual patient medical records and are not publicly available due to patient privacy and confidentiality considerations. De-identified data may be made available from the corresponding author upon reasonable request, subject to applicable institutional and ethical requirements.
